# Beyond the Fellowship: Rebuilding the Pathway to Transplant Leadership

**DOI:** 10.7759/cureus.101610

**Published:** 2026-01-15

**Authors:** Dimitrios Moris, Yuri S Genyk, Emmanouil Giorgakis

**Affiliations:** 1 Transplant, MedStar Georgetown University Hospital, Washington, D.C., USA; 2 Medicine, European University of Cyprus, Nicosia, CYP; 3 Surgery, University of North Carolina at Chapel Hill, Chapel Hill, USA

**Keywords:** competency-based education, hepatopancreatobiliary surgery, surgical leadership, training reform, transplant fellowship

## Abstract

Training in abdominal organ transplantation has historically lacked standardization and alignment with contemporary surgical education paradigms. The current fellowship-based model in the United States provides variable exposure, inconsistent academic structure, and limited integration with related fields such as hepatopancreatobiliary (HPB) surgery and surgical oncology. Drawing lessons from integrated cardiothoracic and vascular surgery pathways, a restructured, competency-based model emphasizing early specialty exposure, academic development, and alignment with national certification frameworks could enhance the quality and coherence of transplant training. Integrated training has been shown to improve recruitment, research productivity, and trainee satisfaction in other disciplines, and similar principles could strengthen transplantation. Moreover, the growing convergence between HPB surgery, oncology, and transplantation highlights the emergence of transplant oncology as a new subspecialty. This evolving field integrates oncologic and advanced HPB surgical principles with core transplant expertise, warranting formal recognition through a dedicated three-year fellowship that includes focused experience in living donor liver transplantation and complex cancer surgery. Reforming transplant education through such models is essential to ensure the sustainability, academic vitality, and multidisciplinary evolution of the field and to reaffirm transplantation’s central role in advancing academic surgery.

## Editorial

Introduction

Few disciplines in modern surgery embody the convergence of technical mastery, physiology, ethics, and innovation as completely as transplantation. However, despite its central role in academic surgery, training in transplantation remains one of the least standardized subspecialty experiences [1]. Across the United States, aspiring transplant surgeons encounter heterogeneous curricula, inconsistent exposure to organ types, and variable emphasis on research and leadership training.

In an era when cardiothoracic, vascular, and plastic surgery have moved toward integrated or competency-based pathways [2,3], the absence of a modern, aligned framework with current and future training paradigms in transplantation risks fragmenting both the workforce and the academic identity of the field. Reforming transplant surgery training is therefore not only an educational necessity but also an ethical obligation to sustain excellence in patient care, research, and innovation.

Any conversation about training reform must begin by acknowledging the national accreditation mechanisms already in place. The Transplant Accreditation and Certification Council now serves as the central body overseeing accreditation of transplant training in the United States. The Transplant Accreditation and Certification Council’s work, standardizing curricula, defining training requirements, reviewing programs, and supporting institutional quality, provides the essential scaffolding for any national pathway. Similarly, the American Society of Transplant Surgeons Fellowship Training Committee continues to lead national efforts in curriculum development, pipeline expansion, mentorship, and competency refinement. With this opinion piece, our goal is not to replace these efforts but to describe how the next stage of training evolution can align with, support, and extend the strong frameworks already established by the American Society of Transplant Surgeons, the Transplant Accreditation and Certification Council, and the Fellowship Training Committee.

Current landscape

To situate proposed reforms accurately within existing oversight structures, it is important to clarify the governance pathway that shapes transplant surgical education in the United States. The American Society of Transplant Surgeons, through its Fellowship Training Committee, develops curricular priorities, educational resources, and competency frameworks that reflect evolving clinical and academic practice. These efforts inform the accreditation and certification functions of the Transplant Accreditation and Certification Council, the primary body responsible for standardizing transplant fellowship requirements, evaluating programs, and ensuring consistency across training sites. Transplant Accreditation and Certification Council, in turn, operates in alignment with broader regulatory and certification environments governed by the American Board of Surgery and the Accreditation Council for Graduate Medical Education, particularly with respect to trainee eligibility, institutional accreditation, and compliance with national educational standards. The models discussed in this manuscript are therefore not proposed as alternatives to existing oversight but as extensions that build upon American Society of Transplant Surgeons-driven educational content, are operationalized through Transplant Accreditation and Certification Council accreditation mechanisms, and remain fully compatible with American Board of Surgery and Accreditation Council for Graduate Medical Education regulatory frameworks.

Transplant surgery fellowships in the United States are primarily accredited through the American Society of Transplant Surgeons, which outlines general expectations for exposure and case mix. However, unlike Accreditation Council for Graduate Medical Education-accredited fellowships, compliance and oversight vary widely. Training characteristics vary, and the combination of organ focus (liver, kidney, pancreas, or multivisceral) and prior hepatopancreatobiliary (HPB) experience determines overall readiness [4]. Moreover, the academic component, including structured education in immunology, clinical research, and outcomes methodology, is inconsistently applied. Surveys indicate that fewer than half feel confident conducting independent research or managing multidisciplinary programs [5]. Representation of women and racial and ethnic minorities among transplant surgeons, and especially in leadership positions, is limited [6,7]. International fellows, who represent a substantial proportion of trainees, face additional barriers to academic integration and certification. This variability undermines the credibility of fellowship certification and creates inequities in competence, research productivity, and career advancement.

The Transplant Accreditation and Certification Council has already laid important groundwork by defining core content, duration standards, and exposure expectations for accredited programs. The American Society of Transplant Surgeons and Fellowship Training Committee have similarly advanced curriculum refinement and pipeline initiatives. Despite these efforts, the pace of change in transplant practice has created gaps between what programs can consistently provide and what the modern field requires. These gaps are most visible in areas such as advanced machine perfusion technologies, normothermic regional perfusion protocols, extended-criteria donor management, transplant oncology, procurement logistics, and even early exposure to fundamentals of xenotransplantation. Many programs offer strong experiences in some of these domains, but variability in institutional volume, technology availability, and regional procurement exposure creates uneven national training outcomes. This variability highlights the need for more structured and coherent integration of emerging competencies across all accredited programs.

Reforming transplant training: core principles

A modernized model for transplant training should provide flexibility while reinforcing the objectives of the Transplant Accreditation and Certification Council and the American Society of Transplant Surgeons. A 4+3 or 5+2 integrated academic pathway could introduce transplant concepts early in residency, fostering identity formation, mentorship, and scholarly engagement. Early immersion in immunology, procurement, and organ allocation would enhance both motivation and technical readiness. Significantly, integrated training does not shorten education but redistributes time toward relevant specialty experiences, aligning with contemporary educational philosophy favoring mastery over repetition (Table 1).

**Table 1:** Comparison of training pathways across surgical subspecialties N/A: not applicable

Specialty	Traditional model	Integrated/competency-based model	Accrediting body	Key features
General surgery	5 years + optional fellowship	N/A	Accreditation Council for Graduate Medical Education	Broad exposure, limited early specialization
Cardiothoracic surgery	5+3	6-year integrated or 4+3 joint	American Board of Thoracic Surgery	Early exposure, improved recruitment
Vascular surgery	5+2	0+5 integrated	American Board of Surgery	Competency-based milestones
Abdominal transplantation	5+2 (American Society of Transplant Surgeons fellowship)	Proposed 4+3 integrated	American Society of Transplant Surgeons/American Board of Surgery (proposed)	Early transplant exposure, academic integration

Evidence from integrated training models in cardiothoracic and vascular surgery provides useful, though not directly transposable, insights for transplant education reform. Since the introduction of the integrated six-year (I-6) cardiothoracic pathway and integrated vascular surgery residencies, multiple studies have demonstrated improved match outcomes, including higher match rates, greater competitiveness, and earlier commitment among highly qualified medical students with strong academic profiles [2,3]. Graduates of integrated pathways have also been shown to achieve operative autonomy earlier, with shorter time to technical independence and comparable or superior board examination performance relative to traditional fellowship-trained counterparts. Significantly, early exposure and longitudinal identity formation within a single specialty have been associated with improved trainee retention, reduced attrition, and stronger alignment between career expectations and eventual practice [2,3]. While transplantation differs fundamentally due to constraints on organ availability, procurement logistics, and multidisciplinary dependencies, the principles underlying these outcomes remain relevant. Early, structured exposure appears to enhance specialty commitment, accelerate competency acquisition, and facilitate deliberate academic development. An integrated or modular academic pathway in transplant surgery, implemented as a pilot within Transplant Accreditation and Certification Council-accredited centers, could reasonably be expected to improve recruitment into transplantation, shorten the transition to independent practice, and enhance retention of surgeon-scientists, provided that safeguards are maintained to ensure broad operative exposure, ethical procurement training, and compliance with existing American Board of Surgery and Accreditation Council for Graduate Medical Education requirements.

The American Society of Transplant Surgeons Core Curriculum represents an important but incomplete step toward national standardization [8]. A mandatory, Accreditation Council for Graduate Medical Education-endorsed national curriculum should define minimum competencies in the technical, clinical, and academic domains. These should encompass procurement and implantation skills, understanding of immunosuppression and organ allocation policy, and training in research methodology, ethics, health economics, and leadership. Implementation should be competency-based rather than time-based, with milestone assessments that align with Transplant Accreditation and Certification Council standards frameworks to ensure comparability across institutions while allowing flexibility in pacing.

Transplantation is fundamentally academic, yet formal scholarly training is rarely embedded within fellowship programs. Additionally, a minority of young transplant surgeons can pursue an academic career and compete for National Institutes of Health funding [9]. Even worse, there is significant gender bias among the minority of young transplant surgeons who pursue an academic career [10]. That is accompanied by significantly less academic productivity in both basic and clinical science [11]. A reformed model should require participation in structured academic pathways that integrate research and leadership development. These might include combined fellowship-Master’s degree programs in Clinical Investigation, Public Health, or Bioethics, dedicated clinician-scientist tracks with protected research time, or structured education and leadership curricula. Embedding such pathways within a national framework would ensure that every transplant fellow graduates with tangible academic credentials and a sustainable career trajectory.

At present, the American Society of Transplant Surgeons accredits programs but not individuals. There is no formal board certification in transplantation through the American Board of Surgery. Establishing a transplant surgery board certification, jointly overseen by the American Society of Transplant Surgeons and the American Board of Surgery, would mirror the successful models in vascular and complex general surgical oncology. Assessment could be anchored in entrustable professional activities, such as performing a liver transplantation as primary surgeon or managing immunosuppression in a multi-organ recipient. National verification of these activities would ensure credibility, accountability, and mobility across systems.

The traditional separation between HPB and transplant training no longer reflects clinical or academic reality. Techniques in vascular reconstruction, biliary surgery, and advanced minimally invasive approaches overlap significantly. An integrated HPB/transplant curriculum would streamline training while producing versatile surgeons capable of addressing the full spectrum of complex HPB and transplant diseases. A modular training structure, with a shared HPB/transplant foundation followed by organ-specific specialization, could promote efficiency without compromising breadth [12]. An emerging concept within surgical education and practice is transplant oncology, which bridges the historically distinct domains of HPB surgery, surgical oncology, and transplantation. Traditionally, HPB surgeons and transplant surgeons have operated within parallel but separate training and practice frameworks, despite increasing overlap in the management of complex liver malignancies. As liver transplantation becomes an integral component of advanced HPB cancer care, these silos must be overcome through integrated training pathways. It may therefore be time to formally recognize transplant oncology as a distinct subspecialty, one that combines advanced HPB surgical training with comprehensive exposure to oncologic principles and transplant immunology. A proposed model could include a three-year transplant oncology fellowship, comprising two years of core transplant training followed by a dedicated year in advanced HPB surgery and living donor liver transplantation. Such a structured pathway would cultivate surgeons equipped to deliver state-of-the-art multidisciplinary care at the interface of oncology and transplantation.

Transplantation also faces a growing workforce crisis. The median age of active transplant surgeons in the United States now exceeds 55 years, while declining interest among residents and procedural complexity threaten the sustainability of the field [13]. Encouraging early exposure through integrated programs, ensuring transparency in outcomes, and expanding pathways for international graduates are essential. Structured national mentorship programs, modeled after the American College of Surgeons and National Institutes of Health frameworks, could pair fellows with mentors beyond their institutions, fostering inclusion, networking, and leadership development.

Academic rationale: why transplantation must lead

Transplantation has always been an academic engine within surgery. The field’s advances in immunology, organ preservation, and outcomes science have shaped modern surgical research culture. Reforming its training system is therefore not a bureaucratic exercise but a reaffirmation of transplantation’s role as a driver of surgical innovation. Modern transplant surgeons must navigate not only operative complexity but also bioethical, policy, and systems-based challenges. They must understand organ allocation equity, engage with emerging technologies such as xenotransplantation [14] and machine perfusion [15,16], and lead multidisciplinary teams in high-stakes environments. Machine perfusion has become central to organ preservation and selection, and trainees must understand the application of normothermic and hypothermic perfusion, dual-perfusion strategies, and viability assessment. NRP has transformed donor management, especially for donation after cardiac death organs, and structured exposure to its protocols, ethical considerations, and technical execution will be fundamental. Similarly, the increasing reliance on extended-criteria and marginal organs requires formal education in evaluating older donors, uncontrolled donation after cardiac death, steatotic grafts, and borderline organs using physiologic, biochemical, and perfusion-based parameters. The trajectory of xenotransplantation also demands attention. As early clinical successes in kidney and heart xenografts accumulate, future transplant surgeons will require foundational knowledge of cross-species immunobiology, bioethics, regulatory structures, and translational science. Finally, the complexity of modern organ recovery systems necessitates formal training in procurement logistics, STAR (strengths, teamwork, alignment, and results) team-style coordination frameworks [17], national allocation practices, and the expanding role of Artificial Intelligence in risk stratification and workflow optimization. These elements are essential not only for technical proficiency but also for leadership in the evolving landscape of organ recovery.

Implementation framework

The proposed integrated and modular pathways are not intended to displace existing Transplant Accreditation and Certification Council-accredited structures. Instead, they are best understood as pilot models that selected centers may adapt to meet evolving competency expectations. Their implementation would require coordination with the American Society of Transplant Surgeons, Fellowship Training Committee, Transplant Accreditation and Certification Council, and, where relevant, regulatory bodies such as the American Board of Surgery and Accreditation Council for Graduate Medical Education. Evidence from early pilot experiences would help determine broader applicability. Clear consensus-building within the transplant community will be necessary before any national adoption, ensuring alignment with accreditation standards and institutional readiness [8]. Selected academic centers could pilot integrated seven-year transplant pathways combining HPB, research, and leadership tracks. Sustainable funding, including institutional and American Society of Transplant Surgeons Foundation grants, should support protected academic time. Longitudinal outcome evaluation would measure trainee satisfaction, academic productivity, and post-graduation competency. Ultimately, these steps could culminate in a formal certification process for graduates of the restructured pathway.

Challenges and counterarguments

Critics may argue that integration risks narrowing early surgical exposure or lengthening overall training. However, experience from cardiothoracic and vascular surgery demonstrates that integrated programs do not reduce general surgical competence and, in fact, enhance recruitment and scholarly output [18]. Others may cite resource disparities, noting that smaller programs lack infrastructure for research or mentorship. National partnerships and virtual curricula can mitigate these inequities, as evidenced by multi-institutional transplant collaboratives that have successfully standardized research and educational content across diverse centers.

These reforms might facilitate the pipeline task force initiatives [19] to increase the interest of general surgery residents in transplant training. In recent years, surveys have demonstrated declining interest in transplantation careers among general surgery residents, with concerns centered on lifestyle demands, intensity of call schedules, and limited perceived work-life balance [20-22].

Conclusions

Transplantation thrives at the intersection of technical excellence and scientific inquiry. To sustain that dual identity, we must reform how we train our future surgeons. Integration, standardization, and academic focus should define the next evolution of transplant surgery education. A reimagined model, built around a unified curriculum, competency-based milestones, and national certification, would not only elevate training quality but also reaffirm transplantation’s place as the intellectual heart of academic surgery. Reforming transplant training is not an option. It is an ethical imperative for the future of our patients, our trainees, and our profession.
